# P-1575. Improving Transplant Infectious Diseases Revenue by Documentation, Coding and Billing Training

**DOI:** 10.1093/ofid/ofaf695.1755

**Published:** 2026-01-11

**Authors:** Courtney E Harris, Laura Sublett, Cassandra Salgado, John E McKinnon

**Affiliations:** Medical University of South Carolina, Charleston, South Carolina; Medical University of South Carolina, Charleston, South Carolina; Medical University of South Carolina, Charleston, South Carolina; Division of Infectious Diseases, Department of Medicine, Medical University of South Carolina,, Charleston, SC

## Abstract

**Background:**

Optimization of documentation, coding, and proper billing can optimize RVU generation for transplant infectious disease groups and allow expansion of services and divisional growth.

**Methods:**

We instituted a documentation, billing, and coding training program at the Infectious Disease division at the Medical University of South Carolina (MUSC) in 2023. The training program was executed for ID providers directing education, billing, documentation, and coding services. Lectures were provided; new documentation templates and correct attestation language were instituted. We addressed changing CMS guidelines. All revenue value units (RVUs) are adjusted for the 2025 CMS reimbursement rate. All billing levels are adjusted to a level 1, 2 and 3 billing scale. Data from 2025 was analyzed for the first 3 months of the year.

**Results:**

Initial consult RVU regeneration increased to 491 RVU for 158 consults in 2022 to 707.66 RVU for 206 consults in 2025. This represented 3.11 RVUs per note in 2022, which increased to 3.44 RVUs per note in 2025. Consult level 3 billing improved from 39.87% in 2022 to 57.48% in 2025. Level 1 notes were eliminated, and level 2 billing was reduced by 26.15% from 2022 to 2025. Similarly, subsequent inpatient notes showed a reduction of level 1 billing from 9.7% down to 2.56% and level 2 from 79.31% to 49.1% and level 3 increase from 10.98% to 48.35%, representing an absolute change of -7.14%, -30.22% and +37.37% respectively. This led to an increased mean RVU per note from 1.39 in 2022 to 1.67 in 2025. For the full year 2024, the adjusted revenue increases compared to 2022 revenue was $18,147 for transplant ID services. In 2025, critical care documentation was started, and G0545 coding was approved for use. The G0545 code has been used in 372 notes, representing 22% of the notes, 331.08 RVUs and $10,710 in revenue for the first 3 months of use.

**Conclusion:**

Transplant infectious disease programs can increase program and divisional revenue by incorporating dedicated training and guidance for proper documentation, coding, and billing. Increased use of critical care billing and G0545 coding will improve overall documentation levels and provide adequate billing for the services provided.

**Disclosures:**

John E. McKinnon, MD, MSc, FIDSA, EMD Serono: Advisor/Consultant

